# Supplementary material for: Towards unified AI-driven fracture mechanics: the extended deep energy method (XDEM)
Source: Nat Commun. 2026 Aug 15;17:8492. doi: 10.1038/s41467-026-76748-1 (PMC13484471; doi:10.1038/s41467-026-76748-1)
Supplement: Supplementary file 3 [file 41467_2026_76748_MOESM3_ESM.zip › pic/Results/Stress_intensity_factor/Mode1/differ_a/crack_detailed_fields06.pdf]

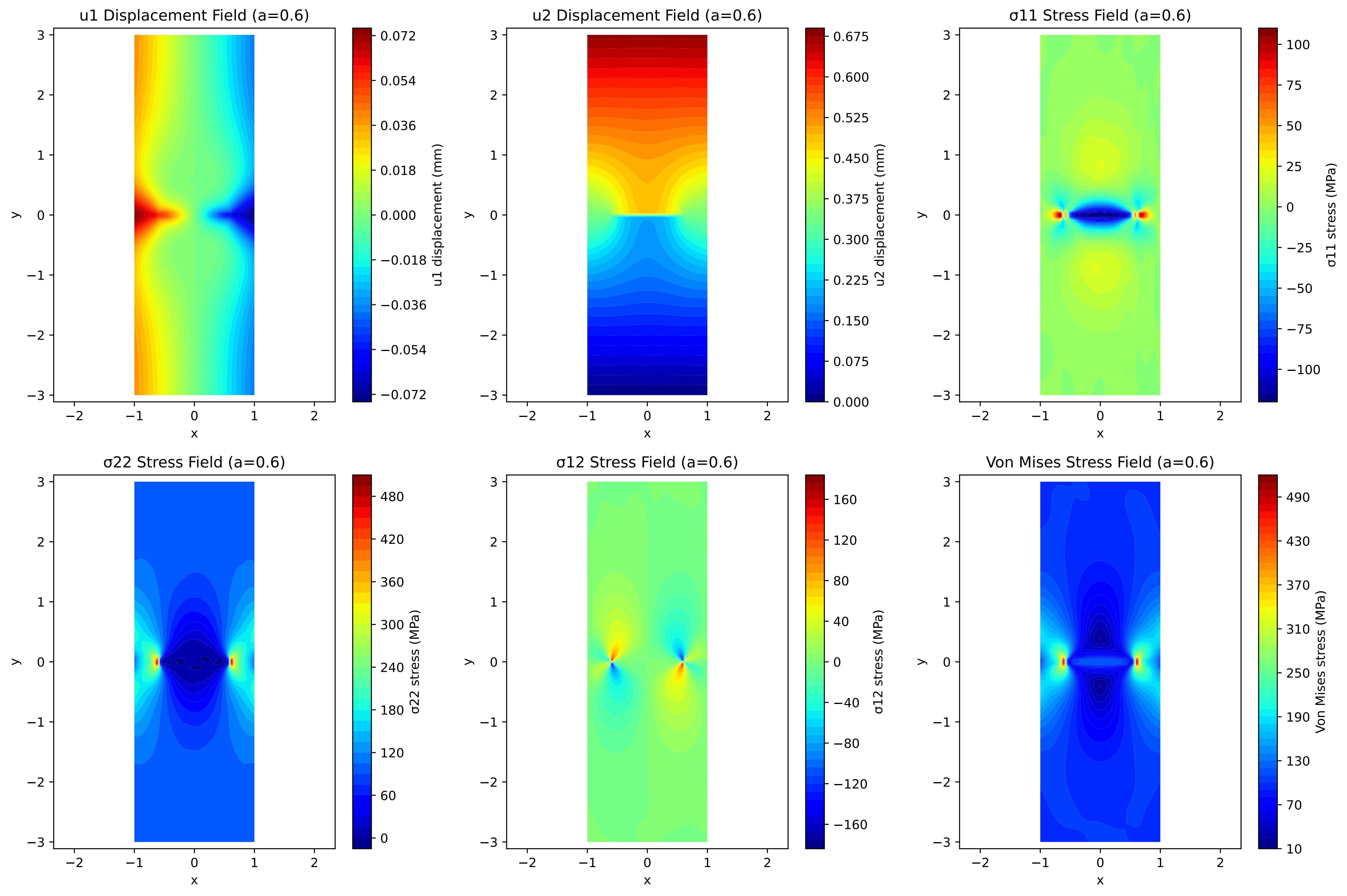

u1 Displacement Field (a=0.6)
u1 displacement (mm)
u2 Displacement Field (a=0.6)
u2 displacement (mm)
σ11 Stress Field (a=0.6)
σ11 stress (MPa)
σ22 Stress Field (a=0.6)
σ22 stress (MPa)
σ12 Stress Field (a=0.6)
σ12 stress (MPa)
Von Mises Stress Field (a=0.6)
Von Mises stress (MPa)
x
y
